# Experimental data manipulations to assess performance of hyperspectral classification models of crop seeds and other objects

**DOI:** 10.1186/s13007-022-00912-z

**Published:** 2022-06-03

**Authors:** Christian Nansen, Mohammad S. Imtiaz, Mohsen B. Mesgaran, Hyoseok Lee

**Affiliations:** 1 Department of Entomology and Nematology, University of California, Davis, USA; 2 Department of Electrical & Computer Engineering, Bradley University, Peoria, USA; 3 Department of Plant Sciences, University of California, Davis, USA; 4 Department of Entomology and Nematology, UC Davis Briggs Hall, Room 367, Davis, CA 95616 USA

**Keywords:** Classification performance, Machine vision, Proximal sensing, Classification models, Seed analysis, Optical sensing

## Abstract

**Background:**

Optical sensing solutions are being developed and adopted to classify a wide range of biological objects, including crop seeds. Performance assessment of optical classification models remains both a priority and a challenge.

**Methods:**

As training data, we acquired hyperspectral imaging data from 3646 individual tomato seeds (germination yes/no) from two tomato varieties. We performed three experimental data manipulations: (1) Object assignment error: effect of individual object in the training data being assigned to the wrong class. (2) Spectral repeatability: effect of introducing known ranges (0–10%) of stochastic noise to individual reflectance values. (3) Size of training data set: effect of reducing numbers of observations in training data. Effects of each of these experimental data manipulations were characterized and quantified based on classifications with two functions [linear discriminant analysis (LDA) and support vector machine (SVM)].

**Results:**

For both classification functions, accuracy decreased linearly in response to introduction of object assignment error and to experimental reduction of spectral repeatability. We also demonstrated that experimental reduction of training data by 20% had negligible effect on classification accuracy. LDA and SVM classification algorithms were applied to independent validation seed samples. LDA-based classifications predicted seed germination with RMSE = 10.56 (variety 1) and 26.15 (variety 2), and SVM-based classifications predicted seed germination with RMSE = 10.44 (variety 1) and 12.58 (variety 2).

**Conclusion:**

We believe this study represents the first, in which optical seed classification included both a thorough performance evaluation of two separate classification functions based on experimental data manipulations, and application of classification models to validation seed samples not included in training data. Proposed experimental data manipulations are discussed in broader contexts and general relevance, and they are suggested as methods for in-depth performance assessments of optical classification models.

## Background

Optical classification and sorting systems are being developed for a wide range of biological objects, including food safety and food quality [1–5], and plant phenotyping and stress detection [6, 7]. Successful development and adoption of optical sensing solutions require complex and highly inter-disciplinary research [8], which has been eloquently described as an “image chain process” [9]. Moreover, each optical sensing solution involves specific research associated with: (1) type of sensor to use, (2) spatial scale at which to acquire optical sensing data, (3) relative uniqueness of object/feature characteristics and classes to divide them into, (4) approaches to data processing, calibration and classification, and (5) market development and economic potential. Despite inter-disciplinary diversity and complexity of optical sensing solutions, there are important denominators with high degree of relevance to all solutions. One of these denominators for systems involving hyperspectral imaging involves methods to optimize image cube segmentation, image correction, and spatial-spectral dimensional denoising [10]. Another denominator with broad relevance to optical sensing systems is the model classification accuracy. In other words, it is the selection of classification function, selection of possible hyperparameters, development and tuning of a classification algorithm, and finally assessment of its overall performance. The latter is the topic of this article. Throughout this article, we focus on optical classification of crop seeds, but we present methods to improve and discussion of performance assessment of classification models with relevance to optical classification of virtually all biological and non-biological objects.

Regarding crop seeds, optical classification systems have been used to assess a number of important quality traits including: classification of near-isogenic crop genotypes and crop classes [11–14], protein content [15–17], moisture content [18–20], mycotoxin levels [21, 22], pathogenic fungi [23, 24], internal defects [25–27], contaminants in seed samples [28], starch content [16], maturity [29, 30], seed weight [16], and viability [31–39]. This range of optical classifications underscores the potential and promise, and there are strong arguments supporting claims that optical sensing systems will in many and important ways revolutionize seed industries in the near future. A recent review on use of hyperspectral imaging in seed quality and safety inspection of seed samples provided comprehensive support for use of this technology to automate and improve grading, defect and disease detection, cleanness detection, composition, and viability/germination of seeds [8]. Facing this considerable potential, it is of paramount importance that researchers developing optical systems to classify seeds and other objects use common/standardized research procedures, so that it is possible to directly compare performance of systems and classification methods. Here, “performance” is considered a composite of two equally important aspects of a given classification model: (1) its “accuracy” (ability to accurately classify objects) and its “robustness” (measured as the classification algorithms inherent sensitivity to stochastic noise) [40]. In their description of model classifications based on neural network functions, Belkin et al. [41] provided an excellent description of classification model development and testing of performance, which was referred to as “generalization”. Classification performance may be asymmetric and therefore show varying degree of ability to classify objects in one or more classes. In cases involving asymmetric classification of objects in two classes, it is often relevant to refer to “sensitivity” (accurate prediction of true positives) and of “specificity” (accurate prediction of true negatives). As part of optimizing performance of classification algorithms, the concept of ‘bias-variance trade-off’ (dilemma) describes the possible trade-off between under- and over-fitting of classification algorithms [41, 42]. High bias (model under-fitting) occurs when classification models are overly simplistic and therefore do not provide an accurate fit to the training data. High variance (model over-fitting) occurs when classification models are overly sensitive and therefore provide model fits to the training data, which include strong effects of stochastic noise in training data. Ideally, a classification algorithm has both low bias and low variance, so that it is sensitive enough to detect subtle differences among similar objects in different classes/categories and ‘robust’ across data sets. As described in recently published articles, neural network functions may be less sensitive to this important trade-off than other classification functions [41]. However, it is well-established that possible classification challenges linked to the bias-variance trade-off are closely linked to the assumption of observations in training sets being random and from a similar probability distribution as those used for model validation [41]. In other words, it is assumed that training and validation data sets follow the same or very similar probability distributions. Data included in this study demonstrate how that assumption can easily be violated. For instance, it is well-known that the same plant genotype grown at different locations and/or under different weather and agronomic conditions can produce seeds or plants with markedly different phenotypic traits (i.e., size, shape, color) [8]. Phenotypic variability, both within and among seed varieties, can markedly decrease performance of optical classification and sorting solutions, as a classification model developed based on a training data set from only one or few subsamples may under-perform when used to classify seeds from other subsamples. Moreover, failure to accurately classify observations from new (validation) samples is likely because the inherent assumption of equal/similar probability distributions was violated. It is therefore critically important to use comprehensive performance assessment procedures to characterize and quantify robustness of a given classification model.

Once developed (i.e., parameters have been tuned), performance assessment of classification accuracy is often based on one of the following validation methods: (1) Jack-knife cross-validation or leave-one-out, in which a single observation is removed from the training data set and used for validation. This method is repeated with all observations to calculate an average classification accuracy. (2) The entire data set is divided into two portions (not necessarily of equal size), in which a data subset is used exclusively to develop a classification model (referred to as training data) while another data subset is used for validation (referred to as validation data). K-fold cross validation is an extension of this method which involves partitioning the full data set into k random portions with k-1 of them being used as training data to generate the classification model, while using the remaining subset for validation. This process is repeated k times and the overall model performance is calculated based on the average of these k repeats. (3) When deep learning models are applied, entire data sets are commonly split into three parts: training, validation, and test data [43]. A combination of training and validation data is used to develop and optimize deep learning models, while a test subset is set aside and used to validate the final classification model. (4) Validation consists of collecting independent validation data (from different locations, different species/variety, optical sensing data acquired on at different time points) to include stochastic noise incurred by both subtle noise/variation in imaging conditions and in preparation of objects being classified. However, limitations of data availability (especially when studies are conducted with animals or plants subjected to experimental treatments), is a common challenge. Thus, due to logistical feasibility constraints, a completely new and in-dependent data set may not be available for classification model validation. It is therefore critical to explore ways to perform thorough classification model performance assessments without additional data.

In all abovementioned validation methods, acquired optical data are divided into training and validation data sets. An alternative or complementary method is to manipulate existing training data by introducing known values or ranges of stochastic noise and/or to alter the size of training data sets. In a recent study, Nansen et al. [44] introduced a method, which is similar to what is referred to as “sensitivity analysis” in population modelling [45]. That is, a classification model is examined based on its sensitivity to each feature/parameter and to levels of stochastic noise associated with these features/parameters in the training data set. We argue that such experimental sensitivity analyses of classification models should be considered more broadly, if the goal is to promote widespread adoption of optical classification models. The main reason being that challenges associated with repeatability [46–48] and/or robustness [11, 40] of classification models applied to optical data have been highlighted as representing some of the most important limitations when developing optical sensing systems. Furthermore, addition of known levels of experimental noise to observations in training data may be considered a way to obtain higher degree of similarity in frequency distributions (and therefore higher degree of classification robustness) between observations in training and validation data sets.

In this study, we describe and discuss methods to examine and quantify performance of optical classification models. As case study, we acquired hyperspectral imaging data from individual tomato seeds, and we developed classification models to differentiate germinating and non-germinating seeds. Performance of classification models was quantified based on ten-fold validation, and we performed three experimental manipulations of training data as a way to thoroughly assess performance of classification models: object assignment error: effect of experimentally assigning 0–50% of tomato seeds to the wrong class. Spectral repeatability: effect of introducing known ranges (0–10%) of stochastic noise to individual reflectance values. Size of training data set: effect of reducing number of observations in the training data set by 0–50%. Accuracy of classification models was also quantified based on independent validation, in which classification algorithms were applied to individual seeds from samples not included in the training data. The main purpose of this study was not to optimize accuracy of the classification results per se but rather to describe and propose methods to minimize concerns about model under- and over-fitting and to propose a repeatable and quantitative approach to performance assessment of classification models.

## Results

**Fig. 1:**
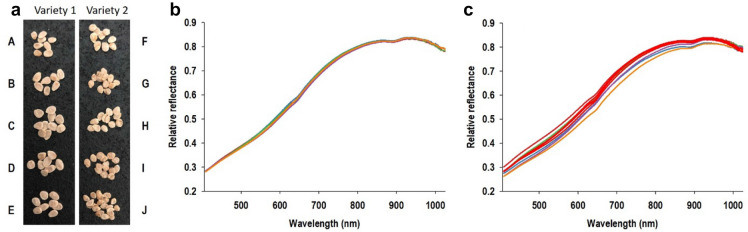
Images and average reflectance profiles of tomato seeds included in this study. Photos of tomato seeds from two varieties, A and B, and five subsamples for each variety (**a**). Average reflectance profiles of five subsamples of tomato seed variety 1 (**b**) and 2 (**c**) included in this study

**Fig. 2:**
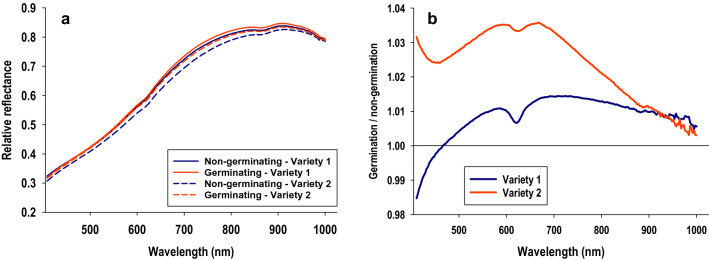
Average reflectance profiles from germinating and non-germinating seeds. Average profiles of non-germinating and germinating tomato seeds from variety 1 and 2 (**a**), and relative effects of germination (germination / non-germination) of variety 1 and 2 (**b**)

Figure 1a shows tomato seeds included in this study from two varieties and with each being represented by five subsamples (seed lots) representing different combinations of growing season and growing location. Although they are virtually indistinguishable by the human eye, average hyperspectral reflectance profiles reveal considerable effects of season and environment based on reflectance profiles (Fig. 1b, c). It is also seen that average reflectance profiles from subsamples of variety 2 were considerably more variable than those from variety 1. For both tomato varieties, similarity of average reflectance profiles from germinating and non-germinating seeds underscores the challenge and also highlights why visual classification by humans would be virtually impossible (Fig. 2a). Figure 2b shows the relative difference between germinating and non-germinating seeds, as average reflectance of germinating seeds was divided with average reflectance of non-germinating seeds. It is seen that germinating seeds had higher average reflectance compared to non- germinating seeds, especially in spectral bands between 600 and 700 nm. It is also seen that differences between germinating and non-germinating seeds were most pronounced for variety 2. Thus in several important ways, this data set represents what is frequently encountered as crucial classification challenges: a high degree of similarity between classes and considerable variation in main treatment effects (in this case, difference between germinating and non-germinating seeds) among subsamples within each class. These common challenges are further exacerbated by factors, such as: (1) error in training data sets (i.e., incorrect assignment of observations to specific classes), (2) spectral noise elicited by non-consistent imaging conditions, and (3) size of training data sets, as it will likely require many observations to accurately separate highly similar classes. Each of these factors were examined as part of this study and are described in separate sections below.

### Assignment error in training data

**Fig. 3:**
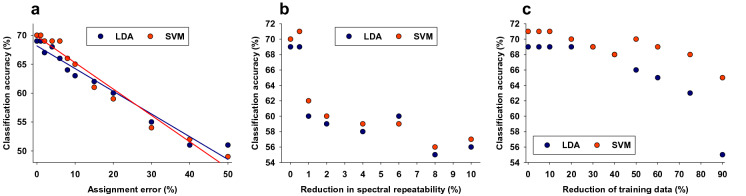
Results from experimental performance assessments of classification models. Training data set from tomato variety 1 was manipulated in three different ways, and for each manipulation, we examined the effect on accuracy of linear discriminant (LDA) and support vector machine (SVM) classification models (based on ten-fold cross validation). Object assignment error: effect of individual seeds being assigned to the wrong class (**a**). Spectral repeatability: effect of introducing known levels of stochastic noise to individual reflectance values (**b**). Size of training data set: effect of randomly reducing the number of observations in the training data set (**c**)

Without experimental mis-assignment of observations, both LDA and SVM classified germinating and non-germinating seeds with about 70% accuracy (Fig. 3a). Based on experimental mis-assignments of 1, 2, 4, 6, 8, 10, 15, 20, 30, 40, and 50% of individual tomato seeds in training data, we observed a negatively linear relationship between level of intentional mis-assignment and classification accuracy (LDA: adjusted R^2^-value = 0.96, slope = − 0.39, intercept = 68.17, SVM: adjusted R^2^-value = 0.96, slope = − 0.46, intercept = 69.81), and the two classification models showed similar responses to mis-assignment of observations. In direct comparison (paired t-test) of LDA and SVM functions, we found no statistical difference in mean classification accuracies (df = 11, t = 1.48, p-value = 0.17). Thus, the two classification functions appeared to have similar tolerance to assignment error.

### Spectral repeatability

Based on experimental manipulation of spectral repeatability (addition of spectral noise to reflectance values in individual spectral bands), we found that LDA and SVM classifications performed similarly with a marked decrease in classification accuracy in response to addition of ranges of spectral noise beyond 1% (Fig. 3b). It is easy to imagine environmental effects on seed and plant growth and/or variations in imaging conditions causing  > 1% change in frequency distribution of optical sensing data from individual objects, so this simple analysis highlights a crucial challenge associated with performance of classification models. In direct comparison (paired t-test) of LDA and SVM functions, we found no statistical difference in mean classification accuracies (df = 7, t = 1.93, p-value = 0.09). Thus, the two classification functions appeared to have similar tolerance to introduction of spectral noise. Importantly, experimental noise ranges added in this study were random and independent among spectral bands, so noise levels could be both positive or negative. Under real-world conditions, it may be expected that spectral noise would show some degree of directionality and not be completely random. That is, difference among imaging events or change during an imaging event in ambient temperatures and humidity, may be expected to cause an overall increase or decrease in intensity of reflectance values. Consequently, it is possible to examine effects of negative and positive noise ranges separately. Also, spectral noise ranges could be added to only portions of spectral bands instead of to all of them, if one spectral region is considered to be more sensitive than others. Thus, there are numerous ways to introduce ranges of spectral noise and to examine their effects on classification accuracy.

### Size of training data set

A major advantage of using seeds in this study was that we were able to obtain large training data sets (Table 1), and we examined effects on classification accuracy by reducing training data sets 5, 10, 20, 30, 40, 50, 60, 75, and 90% for each of the two classes. We found that reducing size of training data sets by 10–20% caused negligible loss in classification accuracy with both LDA and SVM models (Fig. 3c). Regarding SVM-based classification, there was a slight increase in classification accuracy between 40 and 50% reductions of training data. We attribute this slight increase in classification accuracy to random data reductions, which may have led to comparatively more noisy data being excluded. However with both classification algorithms, we observed the expected trend of a negative correlation between training data reduction and classification accuracy. Overall, it is seen that SVM-based classification appeared to be less sensitive to size of training data set than LDA-based classification. An important reason for performing this analysis as part of a performance assessment of a classification model is to determine if obtaining additional training data would improve the accuracy of the model. In this case, we obtained highly consistent classification accuracies with  < 10% data reduction, so it seems reasonable to argue that the potential benefits of adding observations to the training data set would likely be negligible. In direct comparison (paired t-test), we found that the LDA function was significantly more sensitive to reduction of training data compared to SVM (df = 9, t = − 3.18, p-value = 0.01).

**Table 1:** Germination data and numbers of tomato seeds included in this study

Variety and sample	Germination results (%)		Number of seeds	
	Company	Our results	Training	Validation
**1a**	97	97.0, 97.9, 95.8, 92.7	496	96, 96
**1e**	56	56.0, 65.3, 68.8, 58.9	1751	96, 96
1b	83			96, 96
1c	82			96, 96
1d	66			96, 96
**2f**	97	97.0, 94.4, 96.9, 91.7	513	96, 96
**2j**	95	73.0, 74.0, 79.2, 82.7	886	96, 96
2g	91			96, 96
2h	86			96, 96
2i	73			96, 96

Tomato seeds from five subsamples of each of two varieties (1 and 2) were included in this study (10 samples, see also Fig. 1a). Two subsamples for each variety (variety 1: 1a and e, variety 2: 2f and j) were used as training data, and these are highlighted in bold. For all 10 tomato seed subsamples, we obtained germination results (%) from the seed company, and we performed four replicated germination tests of subsamples used as training data. Hyperspectral images of individual tomato seeds in training and validation data sets were acquired on different days, and two sets of validation data were acquired on separate days

### Validation of classification models

**Fig. 4:**
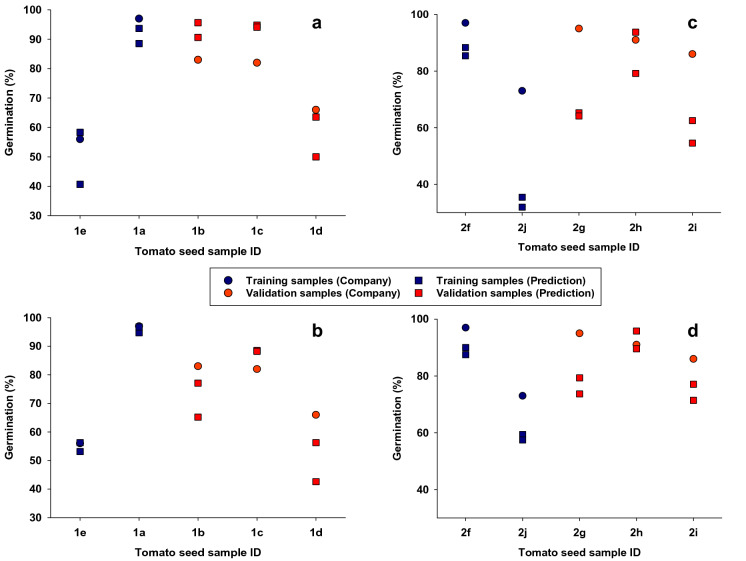
Correlations between observed and predicted seed germination (%) based on linear discriminant (LDA) and support vector machine (SVM) classification models. Validation data (see Table 1) were used to predict tomato seed germination (%) in five seed subsamples from two varieties. We performed validations of both linear discriminant (LDA) and support vector machine (SVM) classification models. Seed germination percentages obtained from the seed company are presented as colored circles and considered “known germination”. Blue circles represent germination percentages of samples, which were used as training data. Red colored circles represent germination percentages of validation samples (not included in training data). Colored squares represent predicted germination percentages of training (blue squares) and validation (red squares) samples. Each colored symbol represents germination percentage based on 96 individual seeds

Figure 4 shows results from validations of classification models, and we present seed germination percentages obtained from the seed company (used as “actual” or “known” germination and presented as colored circles) as well as predictions based on LDA and SVM classification models (presented as colored squares). It is seen that both LDA- (Fig. 4a) and SVM- (Fig. 4b) based predictions of germination of variety 1 tomato seeds were close to germination percentages provided by the company (LDA-RMSE = 10.56 and SVM-RMSE = 10.44, Table 1). Regarding variety 2, LDA-based predictions markedly under-predicted seed germination of subsamples 2j and 2g (Fig. 4c), so the corresponding RMSE was considerably higher than in classifications of variety 1 (LDA-RMSE = 26.15, Table 2). However, SVM-based classifications of germination percentage of variety 2 subsamples were close to germination percentages provided by the company and similar to those obtained in classifications of variety 1 (SVM-RMSE = 12.58, Table 2). Thus, of the two classification functions, SVM-based classification provided the best predictions of germination percentage.

**Table 2:** Root mean square error (RMSE) of validation results

Function	Variety 1	Variety 2
LDA	10.56	26.15
SVM	10.44	12.58

Observed and predicted results from classifications with linear discriminant analysis (LDA) and support vector machine (SVM) functions of validation samples (Fig. 4) from tomato seed varieties 1 and 2

An important side note is illustrated by the four replicated germination percentages in Table 1. That is, we calculated germination percentages from 4 × 96 tomato seeds, and it is seen that results from groups of 96 tomato seeds varied 5–10% points between highest and lowest. This germination range also applies to germination percentages for each seed sample provided by the seed company. As an example, the seed company’s germination percentage for sample 1a = 97.0%, while our replicated tested yielded 97.0, 97.9, 95.8, and 92.7. Thus, any subsample of seeds from 1a might yield a germination result somewhere between 92 and 98%. We mention this, because it seems reasonable to assume that we, at least partially, over-estimated RMSE values (Table 2) as these calculations were based on germination percentages provided by the company (no actual germination percentage data were available for the 20 validation samples of seeds).

### Discussion

Numerous studies describe use of optical classification systems to quantify viability and germination of seeds [31–39]. However, we believe this study represents the first toinclude both a thorough performance evaluation of two separate classification functions based on experimental data manipulations and application of classification models to validation seed samples not included in training data. LDA-based classifications predicted seed germination with RMSE = 10.56 (variety 1) and 26.15 (variety 2), and SVM-based classifications predicted seed germination with RMSE = 10.44 (variety 1) and 12.58 (variety 2). However, the main purpose of this study was not to classify tomato seeds into germinating and non-germinating classes per se but to thoroughly analyze performance of classification algorithms based on three experimental data manipulations. That is, we propose that performance assessments should be adopted, in which training data are experimentally manipulated to specifically assess the classification’s sensitivity to: (1) error in assignment of individual objects to classes, (2) error due to spectral noise (low spectral repeatability), and (3) size of training data set. In the following, we use two published studies to describe the broader relevance of these three data manipulations. That is, we use these two published studies to broaden the discussion about assignment error of observations beyond its relevance to studies of seed classifications.

#### Assignment error—the broader relevance

A recently published study described use of a handheld hyperspectral spectrometer to acquire reflectance values in 2039 spectral bands from leaves of potato plants in each of four treatment classes: non-infected control plants, plants experimentally infected with late blight (*Phytophthora infestans*), plants experimentally infected with early blight (*Alternaria solani*), and plants experimentally infected with both late and early blight [49]. Leaf reflectance data were collected over time, and visual inspection was performed to divide potato plants into four etiological classes Although the etiological stages used to score/rate the severity of each blight disease were both logic and sound, it is clear that they are at least partially subjective and therefore somewhat ambiguous. In experimental studies, plants assigned to a given treatment are occasionally either cross-contaminated and/or appear not to have been treated successfully. Thus, the authors in this study of blight detection stated the following: “*Inoculated samples that did not achieve a disease rating of 4 for their respective diseases or greater by the end of the study period were not included*”. It is always a partially subjective decision for researchers to determine whether or not to exclude observations, and if a considerable number of observations are excluded then the training data may become unbalanced. For instance, in studies with experimental arthropod infestations of plants, non-infested control plants may become infested, so control plants are discarded and disproportionally more so than plants under other treatment regimes. Furthermore, stress symptoms may occur in one portion of a given potato plant, while leaf reflectance data may be acquired from a different portion of the plant canopy. In case of blight and other plant pathogens, there are ample evidence of disease symptoms and pathogens not being uniformly prevalent within individual plants [50, 51]. Thus, in studies involving plants subjected to experimental treatments, it is possible that optical sensing data acquired from leaves were assigned to an incorrect class.

Risk of assignment error exists when optical sensing solutions are being developed on all spatial scales, including airborne hyperspectral imaging. Here, we briefly describe a study on airborne detection and diagnosis of kauri dieback disease [*Phytophthora agathidicida* (PA)] in kauri trees (*Agathis australis*) [52]. A classification of trees was based on vegetation indices, and the training data set consisted of optical data from 1258 reference crowns (tree canopies), which had been divided into five crown classes. Optical classification revealed that random forest classification of reflectance values in six spectral bands showed a 0.93 correlation (mean error = 0.27, RMSE = 0.43) with kauri trees assigned to the numeric dieback scale values from 1 to 5. Thus, this aerial remote sensing study produced a highly accurate classification of objects (trees), but it is highlighted as an example of how assignment of observations/objects may pose challenges and therefore be associated with some degree of error.

The intended take-home message from description of these two studies is that training data sets may include observations assigned to an incorrect class, and likelihood of this object assignment error hinges on number of classes in the training data set,, relative level of distinction (uniqueness) of classes, and expertise and consistency of people performing visual classifications. Through experimental manipulation of a given training data set, individual observations/samples can be assigned to an incorrect class. If the classification algorithm is performing as intended (classifying according to the given trait), then a negative and linear correlation between classification accuracy and number of observations/samples assigned to an incorrect class should be expected. We believe this simple data manipulation method can be readily adopted and used to both assess risk of model over-fitting and to compare performance of different classification models.

#### Spectral repeatability—the broader relevance

Reducing variance associated with average reflectance values is a major challenge in optical sensing, and it is achieved through a wide range of data processing steps, which fall under the general category of spectral calibration [9, 53] to maximize spectral repeatability. Although described in slightly different terminology, a comprehensive review article on use of hyperspectral imaging for seed quality and safety highlighted development and maintenance of calibration models as a major challenge [8]. The main outcome derived from effective calibration models is enhanced spectral repeatability, so calibration models and spectral repeatability are chain-steps in the same process. Concern about spectra repeatability was recently addressed in a study of reflectance profiles acquired from individual beet leafhoppers, in which classification accuracies were compared after adding known ranges of spectral noise to the training data [44]. There at least two general sources of error contributing to low spectral repeatability: (1) inconsistency over time and space of optical features acquired from individual objects, and (2) environmental effects on optical sensing data. Each of these sources is briefly described below.

Inconsistency over time and space of optical features: as an example, we return to the previously described study of dieback disease detection in kauri tree crowns [52]. Importantly, the authors highlighted a number of challenges which could contribute to possible assignment error but especially to lack of spectral repeatability: (1) kauri foliage possess color variations from darker yellow-green to lighter blue-green, which means that some degree of spectral noise is associated with optical data acquired from tree canopies, (2) weakened/stressed kauri trees are more likely to become covered by climbers and epiphytes, which may affect optical data acquired from tree canopies, and (3) adverse growing conditions (i.e., drought, shallow and less fertile soil, and exposure to strong and salty winds from the sea) are known to cause dieback symptoms similar to those induced by kauri dieback disease. As a second example, average reflectance profiles from subsamples of variety 2 were considerably more variable than those from variety 1.

Environmental effects on optical sensing data: even under experimental lab conditions, it is virtually impossible to completely control and maintain truly constant physical conditions. Abiotic conditions affecting consistency of optical data include ambient temperature, humidity, lighting spectrum and intensity, projection angle and distance between lens and target objects. Outdoor, factors including sun angle, cloud cover, shade, scattering from nearby structures/features, atmospheric composition, and wind may also adversely affect repeatability of optical sensing data. In the abovementioned study on detection and diagnosis of late and early blight in potato leaves, the authors stated the following as a way to reduce spectral noise/error: “*Bad measurements, such as those with low reflectance or abnormalities due to measurement error, were removed prior to data analysis* [54–57]”. A decision about “good” and “bad” measurements is of course somewhat subjective and arbitrary, unless it is based on radiometric filtering. That is, it is relatively common to deploy radiometric filters (reflectance thresholds in one or several spectral bands) to automate exclusion of background and “bad” measurements in optical sensing data set acquired with ground [15, 58] or airborne [59] optical studies. Spectral calibration of outdoor optical sensing data may be performed based on deployment of stationary reference objects (empirical line method, ELM) [60] or based on solar and atmospheric modeling (atmospheric radiative transfer models, ARTMs) [53]. Several studies, based on airborne [46] and on ground-based [47, 61, 62] optical sensing data have highlighted spectral noise/error as one of the major challenges when developing accurate and robust classification algorithms. That is spectral repeatability or robustness of optical data are terms used to describe the same challenge—that accurate classification of objects based on optical data relies on reflectance signals varying significantly less among objects within classes than among objects from different classes. Challenges linked to low and inconsistent spectral repeatability, are directly linked to partial violation of the assumption of training and validation data having same or similar frequency distributions. To the best of our knowledge, optimization of spectral repeatability is one of the most important research frontiers for successful and widespread development and adoption of optical sensing systems to classify biological and non-biological objects.

#### Size of training data set—the broader relevance

Regarding optical sensing studies of individual plants or animals subjected to different treatments or in different classes, it is often logistically challenging (practically impossible) to obtain data from hundreds of individual objects. Small sizes of training data sets, constitute a major obstacle in hyperspectral machine vis ion studies, because it can readily lead to model over-fitting when the number of spectral bands (explanatory variables) exceeds the number of observations [63]. “Hughes phenomenon” [64, 65] and the principle of parsimony [63] are terms referring to the same major statistical challenge, which is the risk of model over-fitting [66, 67]. As a general rule and a way to minimize risks of model over-fitting, the following equation has been proposed as a way to calculate the maximum number of explanatory variables (spectral bands) to use [67]:

1 $${\text{Spectral bands}} = \left( {{\text{Objects}} - {\text{classes}}} \right)/3$$

In which, “Objects” is the number of objects/observations and “classes” is the number of treatment classes. As an example, we may have acquired imaging data from 400 seeds in four classes. If so, concerns about possible model over-fitting could be made if > 132 [(400–4)/3 = 132] spectral bands were to be used in the classification of seeds. This issue represents an important (but often ignored) dilemma: that high spectral resolution is perceived as an advantage, as it likely increases to the likelihood of high classification accuracy. However, it also increases risks of model over-fitting and/or increases the need for larger training data sets in order to produce robust classification algorithms. From Eq. 1, it is seen that the number of classes has limited effect on risk of moel over-fitting, provided the number of objects far exceeds number of classes. Otherwise, in studies with few replicated objects and multiple classes, this equation shows that risks of model over-fitting may be eminent. However, Eq. 1 can only be considered a “general rule”, as risks of model over-fitting ultimately depends on the ratio between between-class and within-class variation. Moreover, if objects within each class possess little variation in terms of surface reflectance, and there is high and consistent between-class variation, then concerns about model over-fitting can often be ignored.

There are several ways to address concerns about the ratio between spectral bands and observations. It may be possible to add more observations or to subdivide observations into a higher number, but in most cases reduction of number of spectral bands is the focus and the most feasible method. There are at least four ways to reduce numbers of spectral bands to be included in classification models [9]: (1) exclude spectral bands in specific regions of the radiometric spectrum, if they are considered of low value and/or associated with high degree of spectral noise (low spectral repeatability), (2) through multivariate analyses, such as principal component analyses, convert large numbers of spectral bands into a few axes of variance (principal components), (3) deploy stepwise or feature selection procedures to select spectral bands with high contribution to separation of classes, and (4) through the process of “spectral binning” (averaging spectral bands). Research into feature selection procedures is one of the main frontiers in optical data classification and a wealth of methods have been described and compared [68–71]. As examples of spectral binning, optical sensing data acquired in 240 spectral bands can be averaged × 3 (into 80 spectral bands), × 4 (into 60 spectral bands), or × 5 (into 48 spectral bands). Spectral binning reduces the spectral resolution, which may lead to loss of classification accuracy as discriminatory reflectance responses may be masked. However, there are also examples of how spectral binning increased classification accuracy, as it may reduce error caused by spectral noise [26, 62].

### Conclusions

We believe this study represents the first, in which optical seed classification included independent validation and acquisition of seed imaging data on multiple days and of seed subsamples from multiple combinations of growing seasons and growing locations. However, the main purpose of this study was to propose three experimental data manipulations and showcase how they can be used in performance assessments of classification functions: (1) Object assignment error: effect of individual object in the training data being assigned to the wrong class. (2) Spectral repeatability: effect of introducing known ranges (0–10%) of stochastic noise to individual reflectance values. (3) Size of training data set: effect of reducing numbers of observations in training data. Based on these experimental data manipulations, it was concluded that SVM-based provided the best predictions of germination percentage. Additionally, we demonstrated that LDA-based seed classification was significantly more sensitive to reduction of training data compared to SVM, but the two classification functions show similar sensitivity to addition of experimental spectral noise and to mis-assignment of observations. In broader context and general relevance, the proposed experimental data manipulations may be used in the following manner: if experimental manipulations show, as in the present study, that classification accuracy decreases linearly in response to introduction of object assignment error and that experimental reduction of the training data set by, say < 20%, has only negligible effect on classification accuracy, then we argue that the following important conclusions can be drawn: (1) the given classification model is not classifying noise but is able to detect reflectance features that are associated with optical features directly linked to between-class differences, and (2) the training data set was sufficiently large to ignore important concerns about model over-fitting [62, 63, 66, 67].

## Methods

### Tomato seed samples and germination testing

Ten samples of commercial tomato (*Solanum lycopersicum* L.) seeds were obtained from a seed company, in which five samples were “variety 1” grown at five different locations (referred to as: “1a”–“1e”), and five samples were “variety 2” also grown at five different locations (referred to as: “2f”–“2j”) (Table 1). Due to proprietary nature of these seed samples we are unable to disclose information about the seed samples other than their level of germination (information provided by the seed company): “1a” = 58%, “1b” = 70%, “1c” = 85%, “1d” = 85%, “1e” = 97%, “2f” = 97%, “2g” = 95%, “2 h” = 91%, “2i” = 86%, and “2j” = 73%. Regarding tomato seed samples, “1a”, “1e”, “2f”, and “2j”, we conducted germination assays on individual tomato seeds immediately after they had been subjected to hyperspectral imaging. Individual seeds were placed in wells (volume 250 μL) of 96-well polystyrene assay plates (Thermo Scientific™) filled with 1% agarose (Neta Scientific, Inc.) solution. Plates were sealed with Parafilm^®^ and maintained in a growth chamber (Conviron^®^ GEN1000) at 25 °C. Seed germination, protrusion of radicle > 2 mm, was counted daily for 14 days. Based on linear regression, there was a highly significant correlation between seed germination data obtained from the seed company and those from our own germination tests (adjusted R^2^-value = 0.921, slope = 0.828, intercept = 16.008, F-value = 176.92, P-value < 0.001) (Table 1). Thus, it was considered reasonable to assume that training models based on our germination results from individual seeds could be validated with germination data provided by the seed company.

Tomato seeds from samples “1a” and “1e” constituted training data for classification of germination/non-germination in variety 1 (2247 individual seeds), and “2f”, and “2j” training data for classification of germination/non-germination in variety 2 (1399 individual seeds) (Table 1). Uneven numbers of seeds among training samples were included to maximize the data balance, as samples varied in ratios of germinating: non-germinating seeds. Training data were collected on one day, while validation data were collected on different days. Hyperspectral imaging data acquired from seed samples 1b–d and 2g, h were used as independent validation data (192 individual seeds from each sample, with 96 seed images acquired on two separate days and on days different from those training data were acquired).

### Hyperspectral imaging data acquisition

During acquisition of hyperspectral imaging data from individual tomato seeds, relative humidity was between 30 and 40% and ambient temperature was 19–22 °C. We used a push-broom hyperspectral camera (PIKA XC, Resonon Inc., Bozeman, MT, USA) mounted 20 cm above a moving conveyor belt (2 cm per sec), and hyperspectral images were acquired with a spatial resolution of about 1,500 pixels per seed (frame rate = 150 frames per second). Main specifications of the hyperspectral camera were: digital output (12 bit), and angular field of view of 7 degrees, and objective lens had a 17 mm focal length (maximum aperture of F1.4). Artificial lighting consisted of four 15 W 12 V halogen light bulbs (model = BHD-12V15W, www.amscope.com) on either side of the lens (eight light bulbs) projecting light into an aluminum hemisphere which had been coated on the inside with titanium dioxide. A piece of white Teflon (K-Mac Plastics, MI, USA) was used for white calibration, and the light saturation level was adjusted to the white Teflon, so that radiometric signals were converted into relative reflectance. Hyperspectral imaging data comprise 231 spectral bands from 408 to 1025 nm (spectral resolution = 2.1 nm), but we only analyzed reflectance values in 221 spectral bands from 432 to 1025 nm due to concerns about low signal to noise ratio in the first nine spectral bands.

### Data analyses

Throughout this study, assessment of classification accuracies is based on average result from ten-fold validations. A customized software package was used to acquire individual hyperspectral image cubes from tomato seeds, as they were being imaged on a moving conveyor belt. Data processing and classification were performed in MATLAB (R2020a, Natick, Massachusetts: The MathWorks Inc.) and R v3.6.1 (The R Foundation for Statistical Computing, Vienna, Austria), and all analyses were based on average reflectance profiles from individual seeds. Data from each of the two tomato varieties were analyzed separately. Initial data processing consisted of generating average reflectance profiles from each seed, and associating each seed with its response variable value obtained from germination testing (response variable = “germination”: non-germination = 0 and germination = 1). The R packages, “MASS” and “caret”, were used to perform linear discriminant analyses, LDA [72]. We also used “e1071” R package to perform support vector machine, SVM with linear kernel [73]. No specific hyperparameters were used in any of the classifications (i.e., no alpha0 for LDA and no cost or gamma for SVM). Both LDA and SVM classifications are highly suited methods for binary classifications (such as, germination yes/no), and they have been widely used to classify hyperspectral imaging data acquired from seeds [8]. LDA and SVM classifications of training data from variety 1 were used to address the following three questions:

- Object assignment error: to what extent is classification accuracy affected by potential error in assignment of individual seeds into one of the two response variable classes (germination yes/no)? That is, a common challenge with machine vision classifications is that training data sets rely on some level of supervised classification and error can occur in the assignment of individual objects. As an example, germination was in this study defined as protrusion of radicle > 2 mm, but it is certainly possible that some seeds had a radicle protrusion of slightly below or above 2 mm and therefore were placed in incorrect class. To quantify possible effect of error, we introduced known levels of error/mis-classification into the training data: 0 (no error), 1, 2, 4, 6, 8, 10, 15, 20, 30, 40, and 50%. As a theoretical example: consider a training data set with 2,000 average reflectance profiles of which 1000 were from germinating seeds and 1000 were from non-germinating seeds. Assignment error of, for instance, 6% implies that 60 average reflectance profiles in each of the two classes are selected by random and experimentally assigned to the incorrect class (i.e., non-germinating seeds assigned to germinating seeds and vice versa). The known level of error was assigned randomly to average reflectance profiles, and to ensure balance in the error assignment, it was assigned separately and equally to both germinating and non-germinating seeds.
- Spectral repeatability: to what extent is classification accuracy affected by introduction of known levels of stochastic noise reflectance values in individual spectral bands? That is, spectral repeatability is known as one of the key challenges in applied use of machine vision classifications [11, 40, 46–48], so it is important to characterize how sensitive a given classification model is to stochastic variation in reflectance values in individual spectral bands. Consequently, we manipulated the training data set with 496,145 values (2245 average reflectance profiles × reflectance values in 221 individual spectral bands) and added the following eight ranges of random noise to individual reflectance values (in %): 0 (no spectral noise), ± 0–0.5, ± 0–1.0, ± 0–2.0, ± 0–4.0, ± 0–6.0, ± 0–8.0, and ± 0–10.0. For example, if the average reflectance value in a spectral band for one tomato = 1000 and we experimentally add ± 0–2.0% noise, then the manipulated average reflectance value was a random number between 980 and 1020. Stochastic noise added to one spectral band was independent of that to other spectral bands and also varied randomly among all tomato seeds. Separate model analyses were performed for all combinations of spectral noise range and classification model (LDA and SVM).
- Size of training data set: to what extent is classification accuracy affected by the number of observations (in this case average reflectance profiles from individual tomato seeds) in the training data set? In this study, we analyzed data in 221 spectral bands, and we have tomato seeds in two classes. Surprisingly often, published machine vision studies present results, in which the number of observations was similar or lower than the number of spectral bands i.e. more predictors than observations. Several studies have addressed this performance aspect of classification models [58, 62, 66, 74]. Consequently, to maintain balance between the two classes, we randomly removed: 0% (no removal), 5, 10, 20, 30, 40, 50, 60, 75, and 90% of observations in each of the two classes in the training data. As an example, we may consider a training data set with 2000 observations (1000 for each of two classes). The size of the training data set can be reduced by randomly omitting, for instance, 100 (10%) or 200 (20%) of observations in each of the two classes, and effects of classification performance and be assessed.

Regarding object assignment error and spectral repeatability manipulations, we predicted one of two possible outcomes: (a) association between error and classification accuracy is negative. This outcome would be an indication of the classification model capturing true feature trends in the training data, because it would show evidence of loss of classification accuracy in direct (and possibly linear) response to addition of error. (b) Levels of error and classification accuracy are non-correlated. This outcome would be a strong indication of model over-fitting. Regarding size of training data set, we predicted one of two possible outcomes: (a) association between data reduction and classification accuracy is negative. This outcome would be an indication of the training data set being too small or near the minimum size. (b) Levels of data reduction and classification accuracy are non-correlated. This outcome would be a strong indication of the training data set being sufficiently large. For each of the three data manipulations, we performed paired t-tests (library ggplot2) to compare results with LDA and SVM functions.

The final analysis in this study consisted of applying each of the two classification models to data from 20 validation subsamples of 96 seeds each and with 10 subsamples for each variety (Table 1). For each variety, four validation samples were seeds from the same subsamples used to generate training data, while the remaining six subsamples were from completely independent seed samples. Predicted germination percentages were compared with germination percentages provided by the seed company and used as “known” or “actual” to calculate RMSE-values (library Metrics) for each combination of tomato variety and classification function.

## Data Availability

The datasets during and/or analysed during the current study available from the corresponding author on reasonable request.
